# Pectus Excavatum—A Frequent but Often Neglected Entity in Sports Cardiology

**DOI:** 10.3390/diagnostics15232956

**Published:** 2025-11-21

**Authors:** Łukasz Małek, Anna Lemańska, Mateusz Śpiewak

**Affiliations:** 1 Department of Nursing, Faculty of Rehabilitation, University of Physical Education in Warsaw, 00-968 Warsaw, Poland; 2 Central Clinical Hospital, Medical University of Warsaw, 02-507 Warsaw, Poland; 3 Magnetic Resonance Unit, Department of Radiology, National Institute of Cardiology, 04-628 Warsaw, Poland

**Keywords:** chest deformation, athlete, cardiovascular symptoms, heart disease, pre-participation screening, shared-decision making

## Abstract

Pectus excavatum (PE) is the most frequent chest wall deformity, representing 65–95% of all cases, with an estimated prevalence of up to 1 in 300 births. Despite its frequency, it remains underrecognized in sports cardiology. PE results from sternal depression and narrowing of the anterior chest, which may lead to cardiac compression, impaired diastolic filling, and reduced stroke volume during exercise. Consequently, athletes with PE often present with cardiovascular symptoms such as exercise-induced dyspnoea, chest pain, palpitations, presyncope, or reduced physical fitness. Electrocardiographic changes, including right bundle branch block, axis deviation, atrial enlargement, T-wave inversion, QS complexes or Brugada phenocopies, are frequent and may mimic serious cardiovascular conditions, complicating pre-participation screening. Furthermore, PE is associated with potentially high-risk conditions including mitral valve prolapse, ventricular arrhythmias, and connective tissue disorders such as Marfan syndrome, which carry implications for sports eligibility and safety. Assessment of athletes with PE requires multimodal imaging (echocardiography, computed tomography, magnetic resonance), cardiopulmonary exercise testing, and exclusion of concomitant cardiovascular disease. Treatment strategies range from conservative approaches (physiotherapy, vacuum bell therapy) to surgical correction, most commonly with the Nuss procedure, which can improve cardiac function, exercise capacity, and quality of life. Management should involve shared decision making between clinicians, athletes, and families, weighing potential risks against athletic aspirations. Awareness of PE in sports cardiology is crucial, as it not only influences differential diagnosis and screening outcomes but also impacts career decisions and the psychological well-being of athletes.

## 1. Introduction

Pectus excavatum (PE) or “funnel chest” is the most frequent of all chest deformities accounting for 65–95% cases depending on the reports [1,2]. It is characterized by a smaller distance between the sternum and the spine due to a central depression in the anterior chest wall resembling a funnel. Its prevalence is reported to be up to 1/125–1/300 births, but due to difference in classification this number may be underestimated [2,3]. A large study of autopsies reported the prevalence of 0.13%, while radiologic findings from the Dallas Heart Study demonstrated that it may be present in 0.4 to 5% of the studied population depending on the definition used [4,5].

**Figure 1:**
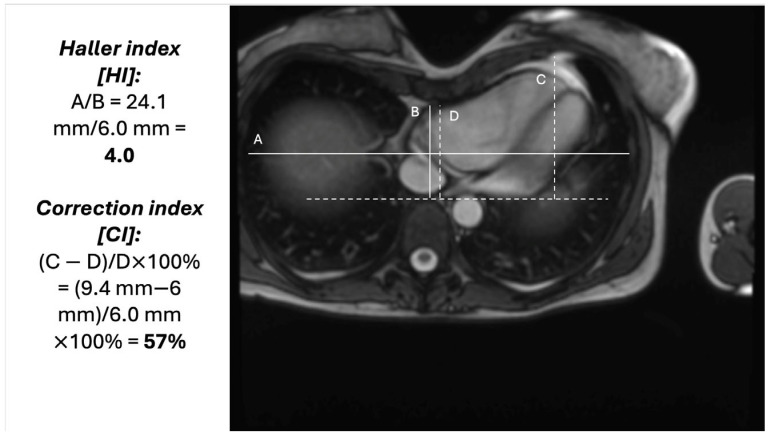
Demonstration of the calculation for Haller index and correction index. Demonstration of the calculation for Haller index and correction index. HI above 3.25 and CI above 28% are an indication for surgery.

Pectus excavatum is more frequent in males than in females (3-4:1) and in Caucasians [1,2]. It is present at birth but usually worsens with age which is related to decreasing chest elasticity, progression of accompanying spinal deformities (scoliosis or kyphosis) and/or the onset of acquired diseases (e.g., chronic obstructive pulmonary disease) [6]. There are various hypotheses on PE origin including retraction of the diaphragm, overgrowth of the anterior chest wall, growth disturbance theory and multifactorial genetic predisposition [1,2]. Apart from the physical examination, the diagnosis in mainly based on imaging studies like computed tomography or magnetic resonance of the chest. The most often used classification measures include the Haller index (HI) and pectus correction index (CI), which are presented in Figure 1, but the full assessment is not limited to those indices and is usually based on other criteria such as sternal torsion, depression index, cardiac compression index and deformity indexes [7,8,9,10,11].

Patients with HI > 3.25 or CI above 28% may become candidates for surgery if PE is accompanied by symptoms, objective cardiac compression, significant arrhythmia, or syncope/presyncope [1,2]. The two main repair procedures are Ravitch procedure and, the more often used minimally invasive repair of PE (MIRPE) called, the Nuss procedure [1,2]. Less severe cases can be treated with rehabilitation comprising vacuum bell therapy, orthosis, posture correction exercises, breathing exercises and regular physical activity [12].

Pectus excavatum may present with numerous mainly cardiovascular (CV) symptoms related to heart compression within smaller chest cavity and its entrapment between the anterior chest wall and the spine. More severe phenotypes are also characterized by left or right sternal torsion related to larger deformation, depression, and asymmetry of the anterior chest wall [9]. Cardiac compression often shifts the heart position leftwards (sinistrocardia) and causes modelling of the right atrium and/or the right ventricle on the anterior wall. This modelling and compression may affect diastolic function of the heart influencing its filling with blood particularly during exercise. Right ventricle (RV) is mainly compromised, but left ventricular diastolic filling can also be impaired secondarily. Pectus excavatum commonly produces extrinsic compression leading to reduced RV end-diastolic volume, altered geometry, and reduced compliance causing impaired RV diastolic filling and reduced stroke volume. Left ventricle (LV) diastolic filling abnormalities are reported in a sizeable minority and LV impairment usually occurs indirectly via reduced RV preload, interventricular dependence, altered cardiac position, or generalized restriction of intrathoracic volume rather than direct anterior compression of the LV [13].

Chronotropic compensation enables patients with PE to maintain the expected exercise cardiac output, but at a cost of reducing ventricular filling time at a given exercise intensity and an earlier attainment of peak heart rate (HR) [14]. For these reasons, patients with PE may report higher HR than expected by intensity of exercise, markedly decreased physical fitness, exercise dyspnoea, and chest pain during exercise. Tricuspid valve (TV) compression and impaired right ventricular filling effecting in lack of adequate stroke volume increase during exercise may even lead to presyncope or syncope [15,16,17]. Right heart friction on the anterior chest wall can be responsible for both atrial and ventricular arrhythmias [18]. Remodelling of the heart to fit in the chest cavity affects also the distribution of pericardial fluid, which may present in the areas of diminished compression such as coronal sulcus, posterior interventricular sulcus and inferior wall of the right and left ventricle. A comprehensive questionnaire (PCAPES) assessing PE symptoms has been proposed [19].

All these symptoms may pose diagnostic difficulties for sports cardiologists at various stages of care of the young athlete from pre-participation screening to competitive sport participation. The most famous athlete affected by PE is Cody Miller, who despite the condition was able to pursue a successful career in swimming leading to numerous Olympic and Championship medals in breaststroke. However, it may not always be the case as the symptoms and physical constraints depend on the specific PE anatomy, sport selection, age, and time. In this review we would like to present the problems which may be encountered by sports cardiologists managing athletes with PE.

## 2. Pre-Participation Screening

Pre-participation screening in young athletes most often include a questionnaire on prior medical history and the presence of symptoms potentially related to CV diseases. It is followed by physical examination (PE) and resting ECG as suggested by current guidelines [20,21].

Athletes with PE may report various symptoms characteristic for CV diseases, for which PE may be the main or only reason as demonstrated in Table 1. Thorough physical examination should be performed to collect information on PE such as depth of the thoracic depression, anteroposterior diameter of the thorax at the same level, and their ratio—anthropometric index (AI). It should also include cardiac auscultation for typical MVP mid-systolic click and late systolic murmur.

Assessment of resting ECG is performed according to international recommendations for electrocardiographic interpretation in athletes [22]. These guidelines divide observed changes into three categories—normal, borderline and abnormal ECG findings. Normal findings are related to physiological, adaptive changes in relation to sport and do not warrant further diagnostics if they are present in asymptomatic athletes with no family history of inherited cardiac disease or sudden cardiac death (SCD). Borderline findings are usually also benign if present in isolation but require further evaluation if there are two or more of them. Finally, abnormal findings always require additional diagnostic work-up.

Patients with PE, due to sinistrocardia and/or heart modelling on the anterior chest wall more often present with ECG findings belonging to all those categories in comparison to normal population [23,24]. These ECG changes include:

- incomplete right bundle branch block (RBBB) which is a normal finding,
- right or left axis deviation, signs of left and/or right atrial enlargement (negative P wave in lead V_1_ or P wave in lead II > 2.5 mm, respectively) or complete RBBB which are all borderline findings,
- negative T waves in V_1_–V_3_, which if not present in black athletes or athletes below 16 years of age, are considered abnormal findings and should always warrant further diagnostics like QS in V_1_–V_4_, mimicking prior myocardial infarction or Brugada phenocopies also found in patients with PE [25,26,27].

Most of these findings are present more often before corrective surgery and decreased significantly after the procedure [23]. Importantly, preoperative presence of RBBB and T wave inversion in leads V_1_–V_3_ were associated with abnormal results in cardiopulmonary exercise testing (CPET) [23]. CPET is crucial in the diagnosis of athletes with PE and exertional dyspnoea as relevant information regarding the cause of this symptom may be obtained with CPET by measuring inspiratory capacity and tidal volume (i.e., VT/IC, signs of dynamic hyperinflation/ventilation-perfusion mismatch as measured by VE/VCO2 slope). It can also be used in differentiation between mechanical vs. cardiovascular limitation, to identify subclinical impairment and to navigate surgical/rehabilitative decision making [28].

Therefore, as demonstrated above, more athletes with PE may require further tests due to the presence of potential CV symptoms and/or suspicious changes in resting ECG (multiple borderline changes or abnormal changes). It may increase the athlete’s anxiety, but also duration and cost of pre-participation screening in this group of athletes. Of course, if PE is clearly visible during physical examination it may be connected to the whole clinical picture and not overlooked. However, if it is less prominent orneglected, the presence of worrying symptoms and/or ECG findings may lead to a false suspicion of CV disease. Some potential scenarios are presented in Table 2.

**Figure 2:**
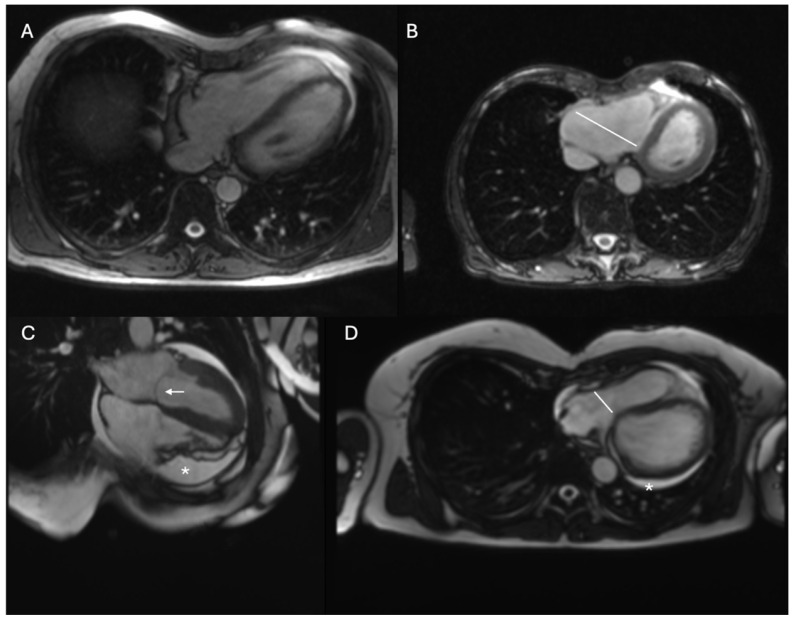
Examples of patients with findings related to the presence of PE. Cine-SSFP pictures from cardiac magnetic resonance (CMR). All patients have PE and heart rotation to the left (sinistrocardia). (**A**)—right ventricular modelling of the anterior chest wall causing chest pain during exercise, (**B**)—right ventricular modelling on the anterior chest wall with ventricular arrhythmia from the RV, marked dilation of the ventricle in the basal portion (white line, 58 mm) and TWI inversion in anterior ECG leads falsly suggesting presence of the arrhythmogenic right ventricular cardiomyopathy (ARVC), (**C**)—pericardial fluid (asterisk) and mitral valve prolapse (arrow), (**D**)—tricuspid valve compression (line, 18 mm) and pericardial fluid (asterisk).

All these situations may lead to temporary or chronic contraindication to physical activity, eliminating athletes from training and competition. Some changes related to PE imitate other diseases leading to false positive findings and unnecessary sport cessation (such as suspicion of arrhythmogenic right ventricular cardiomyopathy [ARVC], Brugada syndrome, previous myocardial infarction, acute pericarditis or constrictive pericarditis or the suspicion of atrial septal defect). Some clinical cases are presented in Figure 2. Other changes such as mitral or tricuspid valve prolapse, exercise induced ventricular arrythmia (EIVA) or aortic aneurysm may accompany PE and may be a true reason for competitive sport elimination.

The first group should be highlighted to reduce the number of referrals for additional tests if PE is present and may be solely responsible for the clinical picture, while the second one requires additional tests due to the presence of potentially high-risk factors related to participation in sports, which is discussed in detail in the next paragraph.

## 3. Potential High-Risk Factors Accompanying PE in Athletes

Pectus excavatum has a multifactorial genetic background and is often also present in close relatives of the affected athletes [1,2]. Importantly, it is also genetically connected with other potentially high-risk conditions in sport. Most frequent of them is mitral valve prolapse (MVP) which itself may lead to numerous CV symptoms. It has been estimated that PE individuals are nearly six times more likely to have MVP than controls [29]. In athletes, benign forms of MVP without the presence of severe symptoms such as pre-syncope or syncope, with only mild mitral regurgitation and no severe arrythmias at rest and during exercise do not alter participation in competitive sports [20,30]. On the other hand, some forms of MVP including moderate to severe mitral regurgitation (MR) and arrhythmogenic predisposition, especially with the presence of EIVA with or without symptoms, may form a contraindication for competitive sports [20,30]. MVP may be accompanied by mitral-annular disjunction (MAD) which is a structural abnormality of the mitral annulus ring with displacement of the posterior mitral valve leaflet towards the left atrium causing the valve to be hypermobile and, if marked, increasing the risk of ventricular arrhythmias [31]. High-risk imaging features accompanying MVP are listed in Table 3 and should always be taken into consideration when assessing an athlete with PE and MVP.

According to guidelines asymptomatic athletes with mild or moderate MR without the presence of risk factors can participate in all forms of competitive sports [20]. Asymptomatic athletes with severe MR without risk factors can engage in low-to-moderate intensity sports after consultation of sports cardiologist if left ventricular diastolic diameter is below 60 mm (or <35 mm/m^2^ in men and <40 mm/m^2^ in women), left ventricular ejection fraction (LVEF) > 60%, resting systolic pulmonary artery pressure (sPAP) < 50 mmHg and they have a normal exercise test result [20]. Symptomatic patients with any of the risk factors should not engage in competitive or recreational sports and should limit their physical activity only to low intensity aerobic exercises [20].

Additionally, apart from MVP pectus excavatum has been shown to be related to higher frequency of exercise induced arrhythmias of supraventricular and ventricular origin [18]. Premature ventricular contractions (PVCs) were recorded in 34.9% of patients with PE and premature supraventricular contractions (sPVCs) in 16.9% of them. Complex forms of ventricular arrhythmia such as non-sustained ventricular tachycardia (nsVT) were noted in 2.6% of patients. Patients at a higher risk of complex arrhythmia had higher Haller index, lower LVEF, and peak ventilatory oxygen consumption (VO_2_peak) in comparison to the rest of the studied population [18]. Any forms of atypical ventricular arrhythmia in athletes and particularly EIVA require further examination (with CMR or electrophysiological study) and if symptomatic, accompanied by structural abnormalities or present in complex forms may become a contraindication to competition unless adequately treated (Table 3) [20].

Athletes with PE also have a higher risk of some connective tissue diseases such as the Marfan syndrome (MFS) and other hereditary thoracic aorta diseases (HTAD, e.g., secondary to Ehlers-Danlos syndrome) [31,32]. Studies demonstrate that Marfan syndrome may be present in up to 5% of patients with PE. Both diseases coexist with MVP but can also lead to aortopathy with ascending aorta aneurysm (AAA) and its rupture. In athletes ascending aorta diameter does not usually increase significantly with training and is not considered as a form of cardiovascular adaptation to exercise [20]. Only 0.3% of young athletes have aortic bulb diameter > 40 mm [33]. Patients with MFS or other HTAD, even with normal ascending aorta diameter, should avoid intensive and very intensive exercises, contact sports, and strength training with preference towards endurance sports. Athletes with MFS/HTAD and moderately dilated aorta (40–45 mm) should engage only in skill sports or mixed/endurance sports of low intensity. If ascending aorta has >45 mm in diameter, athletes with MFS/HTAD should temporarily refrain from sport and competition until corrective surgery [20].

## 4. Influence on Physical Fitness

Apart from symptoms suspicious of CV disease aspiring athletes with PE may complain of reduced physical fitness, especially in endurance and mixed sports, where very high intensity exercises are present. It is in line with scientific reports analyzing physical fitness in this group of patients. Malek et al. reported that patients with PE have lower VO_2_max, a marker of endurance, lower O_2_pulse, a surrogate of stroke volume increase during exercise and low VO_2_max threshold of 41% for increase in lactate accumulation in blood, a measure of anaerobic threshold [34]. The authors concluded that reasons for easy fatigue are related to reduced cardiac and not ventilatory performance. Similar reports were made by other groups [14,35,36]. However, there are also case reports showing that in severe PE cases exercise dyspnoea may be related to hyperinflation due to mechanical restriction of tidal volume expansion rather than cardiac capacity, which may be normal. A lower forced vital capacity (FVC) and forced expiratory volume in 1 s (FEV_1_) at rest are often present in severe cases [17,34].

Successful surgical treatment of PE was shown to subjectively improve respiratory pump efficacy, physical fitness, increase O_2_pulse, and absolute VO_2_max (ml/min) [37,38,39,40,41]. However, not all studies reported post-surgical improvement [42]. A meta-analysis by Media et al. has shown no positive effect of surgical treatment on CPET parameters [43].

## 5. Management of Athletes with Pectus Excavatum

There are no guidelines directly addressed to athletes with PE. Both ESC guidelines in sports cardiology and the new scientific statement from AHA/ACC do not mention PE [19,20]. However, recently, joint specialist societies have published the pectus care guidelines [1].

According to those guidelines all patients with PE should undergo cross-sectional imaging of the thorax to determine the Haller index and review potential compression of the right ventricle. Transthoracic echocardiography (TTE) and lung function testing should be performed to exclude other causes of shortness of breath. Echocardiography can also assess the aortic root and mitral/tricuspid valve. Examination can be challenging in patients with PE due to altered cardiac position and acoustic window limitation. These obstacles can usually be overcome by a choice of more lateral or subcostal apical windows and the use of subcostal four-chamber and RV-focused apical views or employment of right parasternal window if available. Cardiac MRI may be used to support TTE and to assess potential cardiac remodelling. Patients with severe PE and exercise intolerance are recommended to undergo CPET. Cardiopulmonary exercise testing provides useful data to distinguish between cardiovascular limitation, ventilatory limitation, or deconditioning in the evaluation of PE. A VO2max below 85% of predicted is regarded as an abnormality, although in trained athletes with PE this value may be misleading and should not be the only marker of impaired physical fitness. In fact, Casatori et al. have demonstrated that physical activity in patients with PE was a determinant of VO_2_max, whereas it appeared not to affect O_2_pulse related to constrained diastolic filling in PE, which may be, therefore, a better marker of disease severity in athletes [35]. Importantly, every patient with PE must also undergo ocular and musculoskeletal systems assessment wherever there is a history suggestive of a connective tissue disorder.

Patients with Haller index above 3.25 and objective evidence of cardiac compression or symptomatic malignant arrhythmias or syncope/presyncope without any other cause are good candidates for surgery. The document does not directly mention an impaired physical fitness and/or planned athletic career as an indication for surgery, but suggests that there is good evidence that patients who are psychologically impacted by their pectus abnormality benefit from surgery in terms of improved quality of life, reduced depression and anxiety scores, and that the operation has good patient satisfaction [1]. All these psychological effects can be related to impaired physical performance in athletes.

For patients with pectus excavatum, the Nuss procedure and the Ravitch procedure have similar surgical risks and efficacy [1]. The decision as to which operation should be offered should be based on the preference of the patient after a detailed discussion with their surgeon. Surgical correction has been shown to improve right ventricular function as well as signs of relief of cardiac compression. We refer the readers to current guidelines for details and comparisons of the most used techniques, timing of the procedure, potential complications and need for repeat surgery, and differences in surgical technique between pediatric and adult age [1]. Vacuum bell therapy is a safe conservative treatment of PE but results are best for less marked defects and should be started in younger patients [1].

## 6. Practical Summary

**Figure 3:**
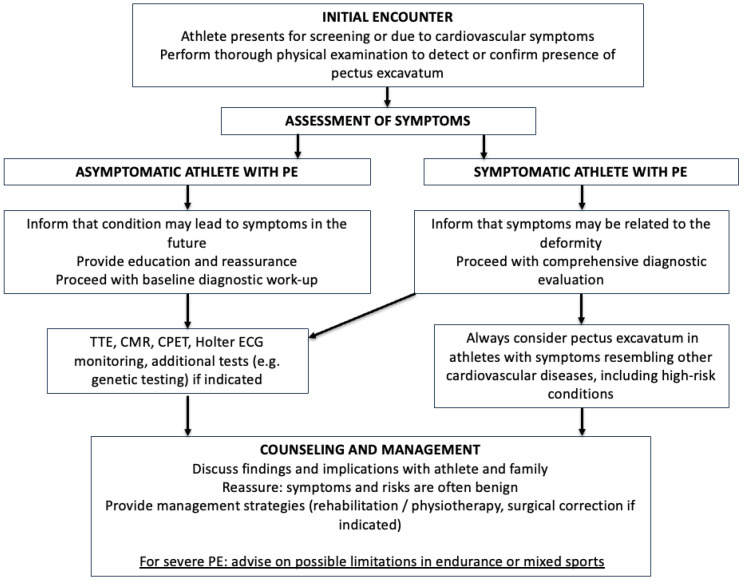
Work-up algorithm for sports cardiologists: evaluation and management of athletes with pectus excavatum.

A practical summary for sport cardiologists, but also for other medical professionals taking care of athletes with signs and/or symptoms of PE is presented Figure 3.

PE should be systematically considered in pre-participation screening to avoid misdiagnosis and unnecessary disqualification of athletes. CV symptoms in young individuals with PE can cause decision making problems and anxiety among athletes and sport medical specialists performing pre-participation screening. Symptoms and signs of PE can mimic other high-risk CV diseases, while diseases accompanying PE form a life-threatening risk for an athlete in case of severe ventricular arrhythmias or syncope. Finally, pectus excavatum can impair a promising athletic career, which should be considered as early as possible and addressed with means of rehabilitation or surgery in the spirit of shared-decision making [20,21,44,45].

## Figures and Tables

**Table 1:** Potential cardiovascular symptoms and diagnostic findings related to pectus excavatum.

Symptoms	Imaging Studies (TTE, CT, CMR)	Other Tests (ECG, Ambulatory ECG, Exercise Test, CPET)
Low physical fitness (easy fatigue)	Sinistrocardia	Negative P wave in V_1_, P wave in II > 2.5 mm, iRBBB/RBBB, TWI in V_1_–V_3,_ QS in V_1_–V_4_, Brugada phenocopies
High resting HR	RA and/or RV modelling on the anterior chest wall	Atrial arrhythmia, also exercise induced
High exercise HR in relation to exercise intensity	Diastolic dysfunction	Ventricular arrhythmia, also exercise induced
Chest pain at rest or during exercise	MVP/TVP	Lower O_2_pulse
Dyspnoea at rest and/or during exercise	Tricuspid valve compression	Lower VO_2_max
Pre-syncope or syncope at rest and/or during/after exercise	Pericardial fluid	
Heart palpitations/rhythm irregularities at rest and/or during exercise	Aortic dilatation	
	Arterial tortuosity	

HR—heart rate, iRBBB—incomplete right bundle branch block, MVP—mitral valve prolapse, RA—right atrium, RBBB—right bundle branch block, RV—right ventricle, TVP—tricuspid valve prolapse, TWI—T wave inversion.

**Table 2:** Potential misleading clinical scenarios in athletes with pectus excavatum requiring differential diagnosis.

Symptoms	Diagnostic Findings	Differential Diagnosis
Palpitations, pre-syncope/syncope	TWI in V_1_–V_3_, ventricular arrhythmias from the RV at rest and/or during exercise, RV modelling	ARVC
Palpitations, pre-syncope/syncope	Brugada phenocopy	Brugada syndrome
Chest pain, easy fatigue	Pericardial fluid	Acute pericarditis or constrictive pericarditis
High resting and exercise HR, easy fatigue	Negative P wave in V_1_, P wave in II > 2.5 mm, ventricular arrhythmias at rest and/or during exercise	POTS, MVP ± MAD, TVP
Chest pain, palpitations	Sinistrocardia	Partial or complete lack of pericardium
Easy fatigue, palpitations	RBBB, RA/RV modelling/enlargement, tricuspid valve compression	ASD, PAPVR

ASD—atrial septal defect, ARVC—arrhythmogenic right ventricular cardiomyopathy, HR—heart rate, MAD—mitral-annular disjunction, MVP—mitral valve prolapse, PAPVR—partial anomalous pulmonary venous return, POTS—postural orthostatic tachycardia syndrome, RA—right atrium, RV—right ventricle, TVP—tricuspid valve prolapse, TWI—T wave inversion.

**Table 3:** Risk factors in athletes with pectus excavatum and accompanying conditions.

Mitral Valve Prolapse	Ventricular Arrhythmia	Aortic Dilatation
TWI in inferior leads	Atypical/high-risk morphology (not LBBB inferior axis or RBBB with QRS < 130 ms), particularly of mitral annular and papillary muscle origin if caused by accompanying MVP	Signs of Marfan syndrome in dedicated questionnaire or signs of Ehlers-Danlos disease
Long QTc in resting ECG	Symptoms	Family history of ascending aorta aneurysm
Bi-leaflet prolapse	Other ECG abnormalities	Hypertension
Severe MR	Persistence or increase during exercise	BAV
Severe systolic LV dysfunction	Polymorphic, repetitive (couplets, triplets or nsVT)	Aortic dilatation >39 mm in female athletes and >40 mm in male athletes
Family history of SCD	Family history of premature SCD or cardiomyopathy	
Documented arrhythmia	Other imaging abnormalities (f.e. LGE on CMR)	
Long MAD	Short coupling	
LGE in infero-lateral LV segment on CMR		

BAV—bicuspid aortic valve, CMR—cardiac magnetic resonance, LBBB—left bundle branch block, LGE—late gadolinium enhancement, LV—left ventricular, nsVT—non-sustained ventricular tachycardia, MVP—mitral valve prolapse, MAD—mitral-annular disjunction, MR—mitral regurgitation, RBBB—right bundle branch block, SCD—sudden cardiac death, TWI—T wave inversion.

## Data Availability

No new data were created or analyzed in this study. Data sharing is not applicable to this article.
